# Colon Perforation Caused by a Transanal Drainage Tube After Robot-Assisted Low Anterior Resection: A Case Report

**DOI:** 10.7759/cureus.98916

**Published:** 2025-12-10

**Authors:** Hiroyuki Hazama, Kohei Koido, Kazumasa Nakamura, Takeshi Oshima, Kou Ohata

**Affiliations:** 1 Gastrointestinal Surgery, Shizuoka General Hospital, Shizuoka, JPN

**Keywords:** colon perforation, iatrogenic injury, low anterior resection, rectal cancer, robot-assisted surgery, transanal drainage tube, tube-related complication

## Abstract

Transanal drainage tubes (TDTs) are commonly used after low anterior resection to reduce intraluminal pressure around the anastomosis. Although associated complications are usually minor, bowel perforation related to TDT placement is exceedingly rare. We report the case of a 71-year-old man who underwent robot-assisted laparoscopic low anterior resection for rectal cancer. A TDT was inserted intraoperatively, and its position was adjusted under laparoscopic guidance. Abdominal radiography performed on postoperative day (POD) 1 showed that the tube tip was located approximately 7 cm proximal to the anastomosis. On POD 4, the patient developed abdominal pain and signs of peritonitis. Computed tomography demonstrated free air and localized fluid collection near the tube tip. Anastomotic leakage or TDT-induced bowel perforation was suspected, and emergency reoperation was undertaken. Laparoscopic exploration confirmed that the TDT had penetrated the colon wall proximal to the anastomosis, resulting in a perforation. Primary suture repair of the perforation and creation of a diverting loop ileostomy were performed. The postoperative course was uneventful, and the patient recovered without further complications. This case highlights an uncommon but serious complication associated with TDT placement and underscores the importance of meticulous intraoperative positioning, close postoperative monitoring, and prompt evaluation when unexpected abdominal symptoms arise.

## Introduction

Anastomotic leakage (AL) remains one of the most critical complications following low anterior resection (LAR) for rectal cancer. Transanal drainage tubes (TDTs) have been used in some institutions with the intention of reducing intraluminal pressure and protecting the anastomosis, although their clinical value remains controversial. The commonly cited mechanism, partial relaxation of the anal sphincter and continuous decompression of the rectal vault, has been challenged, and practice patterns differ substantially across regions. In particular, intraluminal tubes are generally not recommended in the United States, where their theoretical benefits are considered unproven, and concerns about device-related harm have been raised.

Conversely, several recent studies from Asian and European cohorts have reported that TDT placement may reduce the risk of AL in selected patients. A randomized clinical trial by Zhao et al. demonstrated significantly lower AL rates after laparoscopic LAR with TDT use [1]. A network meta-analysis by Yeow et al. similarly suggested potential benefit over no drainage or diverting stoma [2], and a recent systematic review by Tamura et al. further supported the possible effectiveness and safety of TDTs [3]. However, these findings are not universally accepted, and the overall quality and generalizability of the evidence remain heterogeneous.

Although most reported adverse events are minor, such as discomfort or bleeding, rare but severe complications, including bowel perforation, have been described. Hiraki et al. and Sato et al. reported colonic perforations associated with excessive insertion depth or tip-induced pressure injury [4,5]. The present case describes a similarly rare but preventable complication following robot-assisted LAR, highlighting the importance of cautious placement and postoperative evaluation whenever a TDT is used.

## Case presentation

This case report has been prepared in accordance with the SCARE 2025 criteria [6]. A 71-year-old man was referred to our department after a colonoscopy performed for diarrhea revealed advanced rectal cancer. His medical history included hypertension diagnosed at age 67 and a left ankle fracture at age 70. His father had a history of gastric cancer.

Colonoscopy demonstrated a circumferential type 2 tumor measuring approximately 50 mm in diameter, located 8 cm from the anal verge. Biopsy revealed a well-differentiated adenocarcinoma. Preoperative imaging showed no distant metastasis, and the clinical stage was cT3N1M0, stage IIIB (UICC TNM 8th edition). No neoadjuvant therapy was administered.

Robot-assisted laparoscopic low anterior resection with total mesorectal excision and bilateral lateral pelvic lymph node dissection was performed using the double-stapling technique, in accordance with Japanese guidelines for lower rectal cancer. The colorectal anastomosis was located 5.5 cm from the anal verge. Before anastomosis, indocyanine green (ICG) fluorescence angiography confirmed adequate perfusion of both the proximal and distal bowel segments, and after completion of the anastomosis, an intraoperative air-saline leak test demonstrated no evidence of air leakage. After anastomosis, a transanal drainage tube (TDT; Deupul Drain SP®, Create Medic Co., Ltd., Kanagawa, Japan; PMDA approval number: 306ADBZX00011000) was inserted. This tube is a straight silicone device with a total length of 40 cm, an outer diameter of 24 Fr (8 mm), and a 3-cm tip with eight radial drainage slits. The distal tip was manually modified intraoperatively by making fine scissor incisions to create a more flexible octopus-shaped configuration in an attempt to reduce rigidity and potential mucosal pressure.

The insertion length was determined intraoperatively under laparoscopic visualization so that the tube tip passed beyond the anastomosis without bending and without direct contact with the bowel wall. The tube was secured by passing a suture through the perianal skin and tying it directly around the drain. The external portion of the tube was cut 4 cm from the fixation site to allow free drainage. The insertion and position confirmation were performed by another surgeon who was not part of the operative team, and no intraoperative measurement of the insertion length from the anal verge or anastomosis was made. An additional closed-suction drain was placed from the right lower quadrant toward the posterior aspect of the anastomosis. The operative time was 244 minutes, and blood loss was 5 mL. The immediate postoperative course was uneventful.

**Figure 1:**
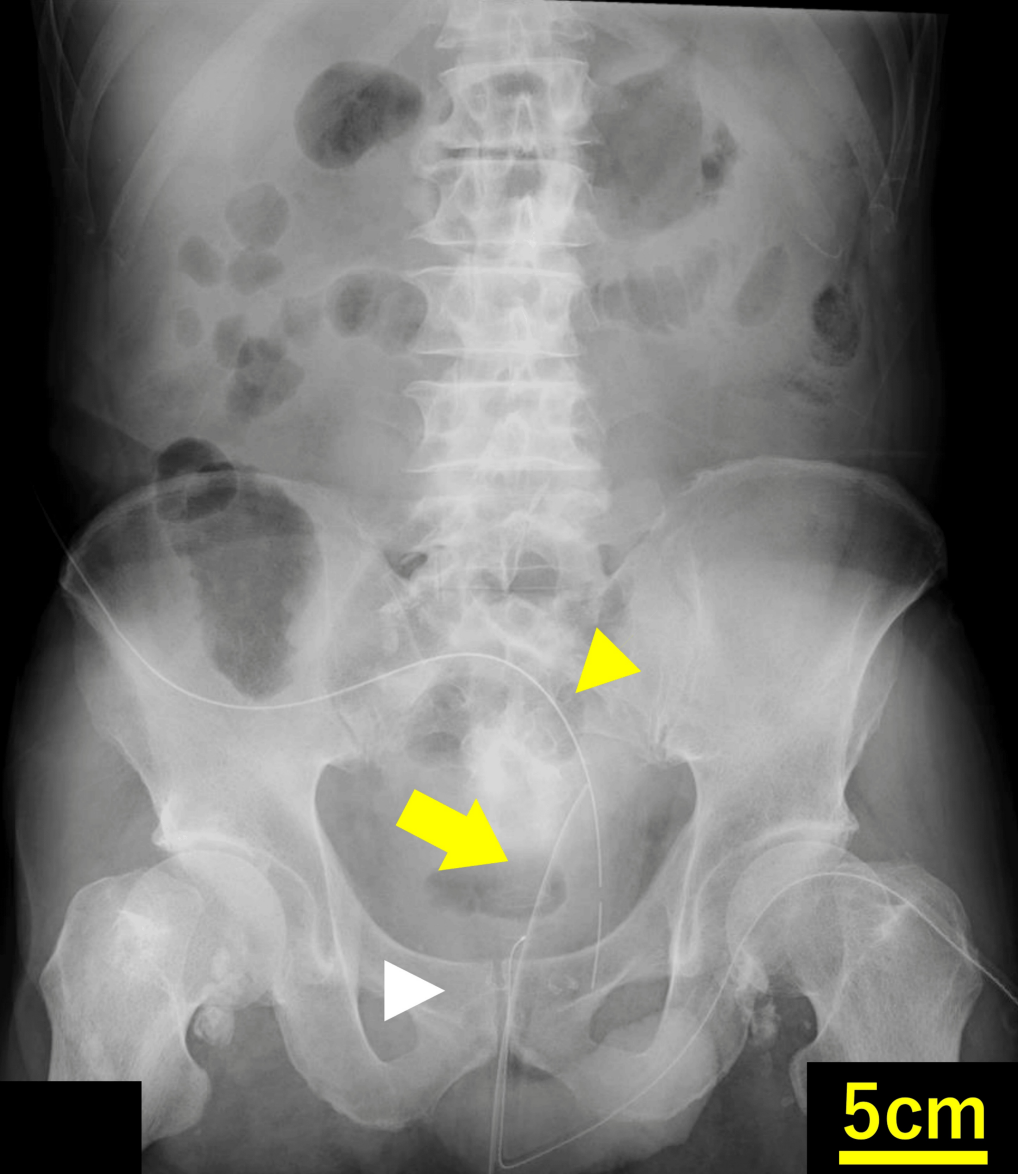
Abdominal radiograph on POD 1 showing the transanal drainage tube (yellow arrow), the anastomotic drain (yellow arrowhead), and the anastomotic site (white arrowhead).

On postoperative day (POD) one, abdominal radiography confirmed the positions of the TDT and the anastomotic drain (Figure 1).

**Figure 2:**
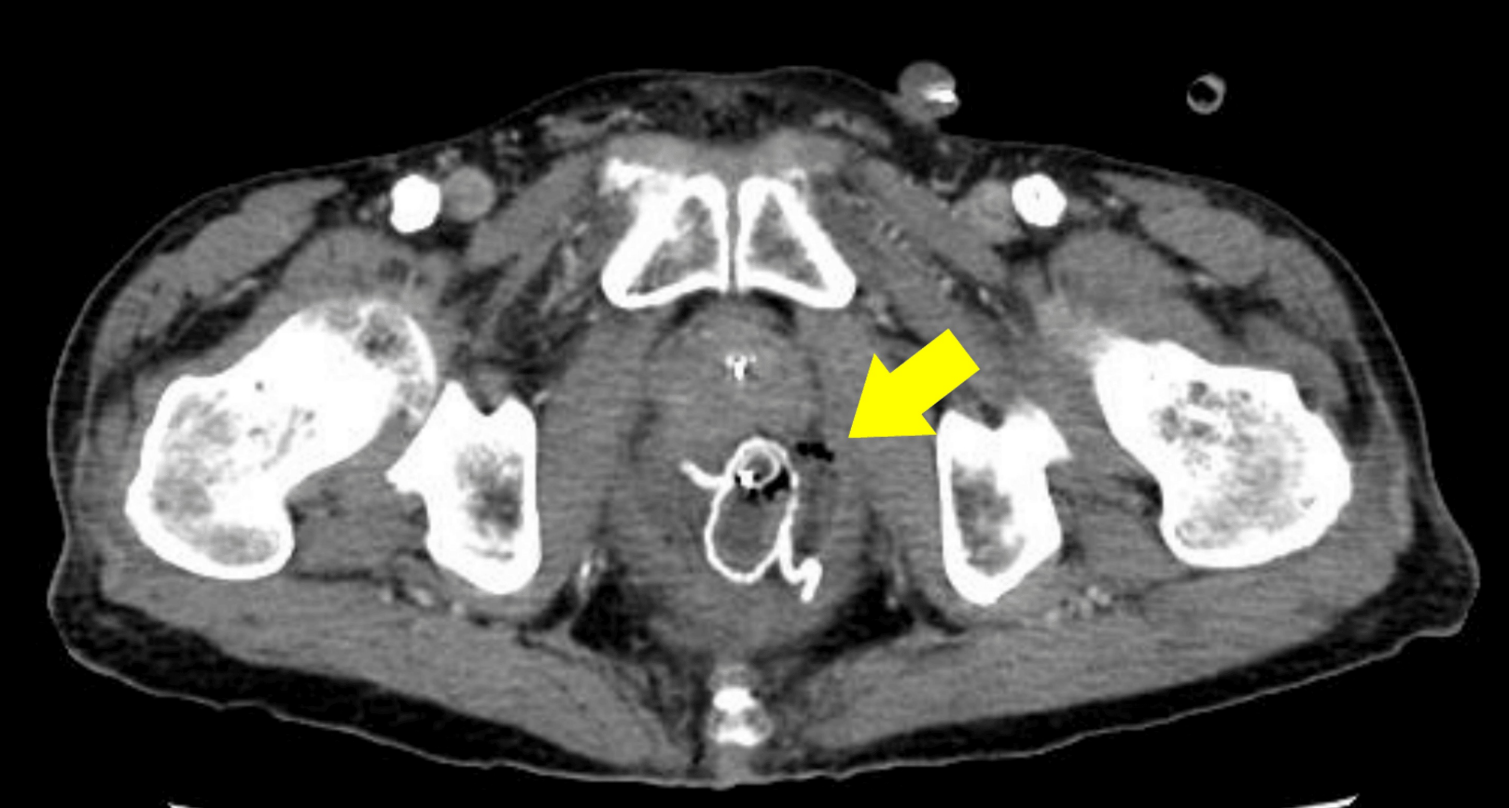
Contrast-enhanced CT. Staple line continuity at the anastomosis is preserved, but perianastomotic fluid and free air are present (yellow arrow).

On POD 2, the patient developed a fever (37.6°C), abdominal distension, and mild abdominal tenderness. Laboratory tests showed a white blood cell (WBC) count of 7,000/µL and a C-reactive protein (CRP) level of 22.24 mg/dL. The anastomotic drain effluent was serosanguineous without debris, while the TDT produced bloody mucus. Contrast-enhanced computed tomography (CT) (slice thickness, 5 mm; portal venous phase) demonstrated preserved staple line continuity at the anastomosis but revealed a small amount of perianastomotic fluid and free air (Figure 2).

**Figure 3:**
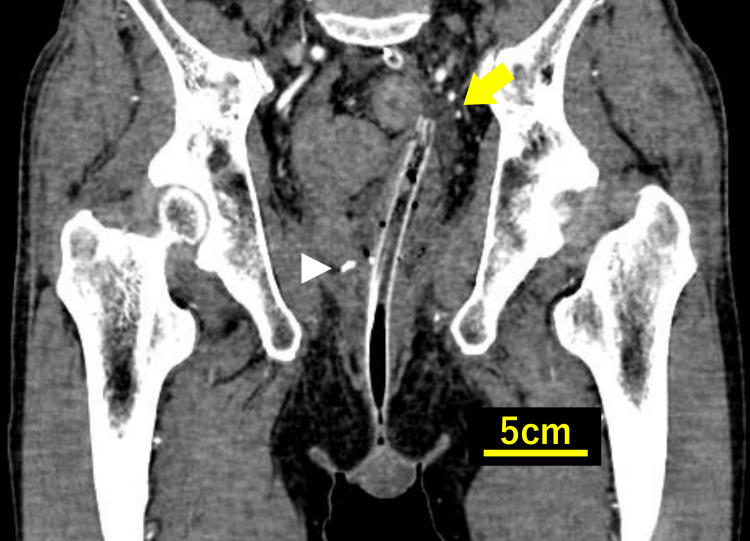
Contrast-enhanced CT image (coronal view) showing the transanal drainage tube (yellow arrow) deviating to the left and slightly penetrating the bowel wall. The anastomotic site is indicated by a white arrowhead. (CT slice thickness: 1 mm, portal venous phase).

On coronal images, the TDT deviated slightly to the left and was located at the level of the bowel wall approximately 7 cm proximal to the anastomosis, corresponding to about 12 cm from the anal verge, with an appearance suggestive of wall penetration (Figure 3).

**Figure 4:**
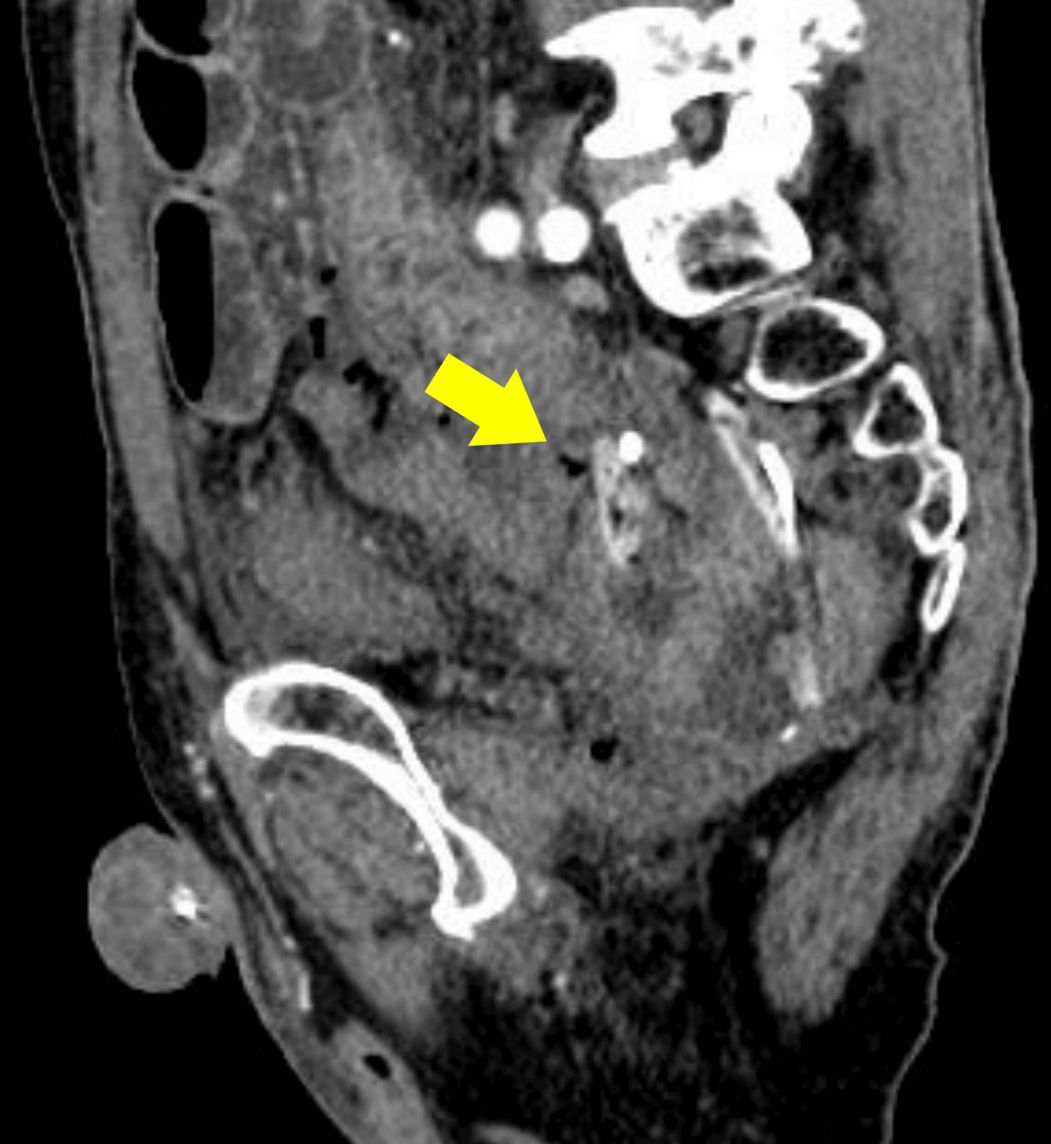
Contrast-enhanced CT image (sagittal view) showing small amounts of fluid and free air around the tip of the transanal drainage tube (yellow arrow), suggesting localized perforation. (CT slice thickness: 1 mm, portal venous phase).

Sagittal images showed fluid and free air surrounding the tube tip (Figure 4).

Based on these findings, anastomotic leakage or bowel perforation involving the segment where the TDT was located was suspected, and reoperation was undertaken.

**Figure 5:**
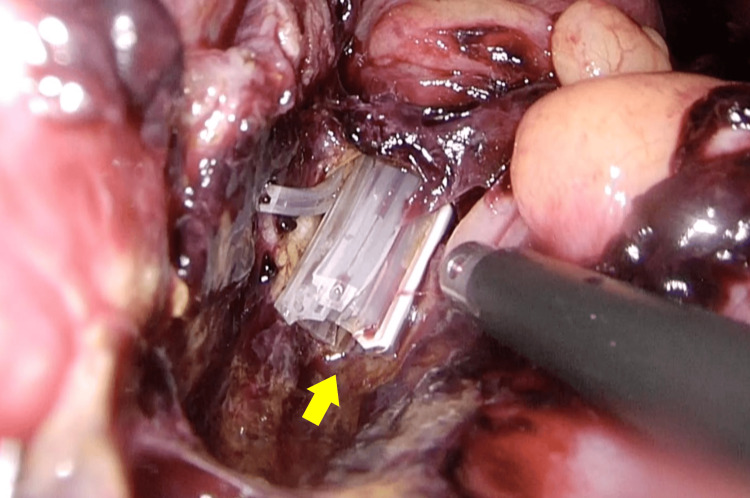
Laparoscopic findings at reoperation showing the TDT tip penetrating the bowel wall with feculent contamination (yellow arrow).

Laparoscopic re-exploration revealed scattered blood clots but no contaminated ascites. The TDT tip was located near the left pelvic wall, and feculent contamination was observed in the surrounding area (Figure 5).

**Figure 6:**
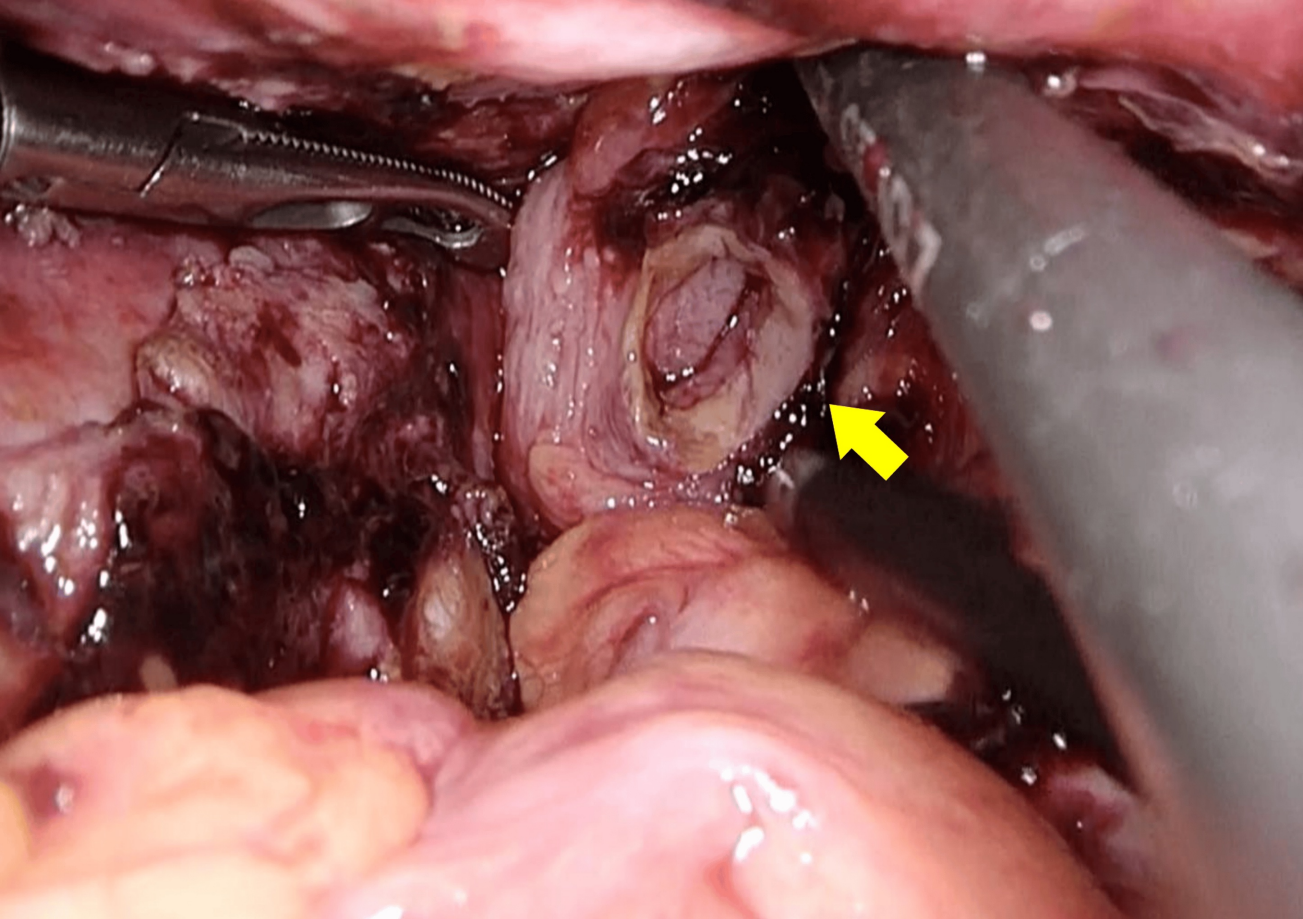
A perforation corresponding to the tube diameter was identified (yellow arrow).

**Figure 7:**
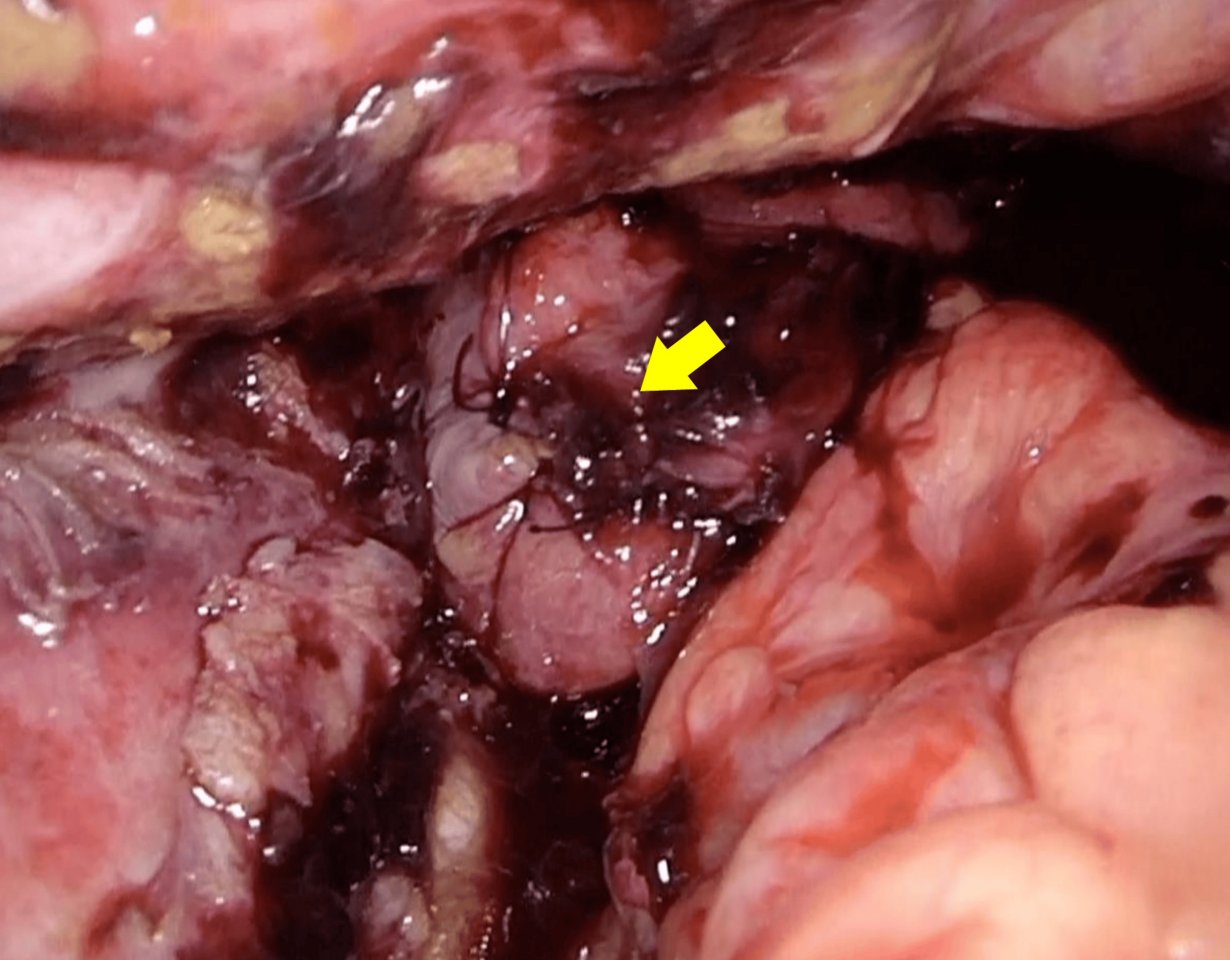
The perforation was closed laparoscopically with interrupted sutures (yellow arrow).

After removal, a sharp perforation corresponding to the tube diameter was identified (Figure 6), while the anastomosis itself was intact. The perforation site was closed laparoscopically with interrupted seromuscular sutures using 3-0 absorbable material (Figure 7).

No bowel resection was performed. Four additional drains were placed around the anastomosis and in both subphrenic spaces.

Although the anastomosis remained intact, a diverting loop ileostomy was created to minimize the risk of intra-abdominal contamination and to protect the repaired perforation under potentially septic conditions. The TDT was not reinserted. The operative time was 131 minutes, and blood loss was minimal.

The postoperative course was uneventful. Oral intake was resumed on POD 4, and the patient was discharged on POD 13 after reoperation. According to the Clavien-Dindo classification, this complication was graded as IIIb. The pathological diagnosis was pT3N0M0, stage IIA (UICC TNM 8th edition). No adjuvant chemotherapy was administered. At three months postoperatively, colonoscopy, contrast enema, and CT confirmed the absence of recurrence or leakage. Ileostomy closure was performed four months after reoperation.

## Discussion

This is a rare case of bowel perforation caused by a transanal drainage tube (TDT) after low anterior resection for rectal cancer. Although the tube was believed to be appropriately positioned intraoperatively, computed tomography (CT) performed at symptom onset revealed that the TDT tip had migrated to a location approximately 7 cm proximal to the anastomosis.

Multiple randomized trials and meta-analyses have suggested that TDT placement may reduce the risk of anastomotic leakage by mechanisms that include decreasing intraluminal pressure and maintaining partial relaxation of the anal sphincter [1-3]. However, these physiological concepts have been questioned, the overall quality and generalizability of the evidence remain heterogeneous, and current practice varies across regions, with some centers, particularly in the United States, refraining from routine intraluminal tube use.

Several case reports, including those by Hiraki et al. and Sato et al., have described bowel perforations associated with TDT use [4,5]. In many of these reports, the tube had been advanced 15-20 cm or more from the anal verge, and/or the tip was clearly angulated or impacted against the bowel wall, leading to focal pressure necrosis. Reported mechanisms include excessive insertion depth, tip rigidity, shearing forces from angled positioning, and insufficient external fixation.

In contrast, in the present case, the tube tip was located approximately 7 cm proximal to the anastomosis, which, although seemingly within an acceptable range, may have produced a hazardous configuration within the narrow pelvic cavity when combined with postoperative peristalsis, changes in patient posture, and the chosen fixation method. Thus, the perforation may have resulted from a problem of relative depth and spatial relationship rather than extreme numerical insertion length alone.

In our procedure, the insertion length from the anal verge was determined visually but was not numerically measured or documented, and the tube was fixed to the perianal skin with a single suture and a 4-cm longitudinal cut in the external segment, a configuration that may have allowed axial sliding despite appearing externally secured. Although radiographic assessment was performed on postoperative day (POD) 1, plain radiography already suggested that the tube had deviated toward the pelvic sidewall; the possibility that excessive advancement might cause perforation was not fully recognized, and tube shortening was not attempted. Postoperative bowel peristalsis and patient movement may further alter the angle or depth of a TDT, resulting in focal pressure necrosis that cannot be detected on static radiographs. These considerations underscore the importance of active postoperative management rather than simple confirmation of tube placement.

From a diagnostic perspective, certain imaging features may help differentiate primary anastomotic leakage from TDT-related perforation. In our case, contrast-enhanced CT demonstrated preservation of staple line continuity at the anastomosis, whereas a focal collection of fluid and free air was observed around the tube tip at a site proximal to the anastomosis. This pattern is more suggestive of a tube-related perforation than of a primary anastomotic leak, in which discontinuity of the staple line and a broader perianastomotic collection centered on the anastomosis are more typically seen. These considerations may aid clinicians in selecting the most appropriate re-intervention strategy when early postoperative deterioration occurs in patients with a TDT in place.

To promote safer TDT use, we propose the following pragmatic, literature-informed recommendations: 1) Limit insertion depth to a few centimeters beyond the anastomosis. Excessive advancement (particularly beyond approximately 10-12 cm from the anal verge) has been associated with pressure injury and perforation [4,5]. 2) Confirm and document tip position intraoperatively and early after surgery (POD 0-2). If the tube is excessively deep or kinked, immediate shortening or removal should be considered. 3) Restrict the duration of placement to within 5-7 days. Prolonged retention has not shown additional benefit and may increase mucosal injury risk [1]. 4) Use soft, atraumatic silicone tubes and ensure fixation that minimizes axial sliding. Because fixation often relies on perianal skin sutures alone, care should be taken to avoid situations (such as prolonged sitting) that may push the tube inward, and external markings or supportive dressings can be used to maintain a constant external length. In Japan, octopus-type silicone tubes with multiple slit openings are commonly used, and further refinement toward a softer, more rounded, atraumatic design may help reduce mucosal injury. 5) Consider a diverting ileostomy instead of TDT placement in high-risk anastomoses (low rectal, irradiated, tensioned, or borderline-perfused sites). A diverting ileostomy remains the most reliable strategy to reduce bowel distension and mechanical stress on a vulnerable anastomosis. 6) Prompt reassessment with contrast-enhanced CT or endoscopy should be performed if concerning findings arise in the early postoperative phase, including new or worsening fever, progressive abdominal distension, localized abdominal tenderness, a marked rise in inflammatory markers (particularly C-reactive protein), or changes in the character of drain output. When such signs appear on postoperative days 1-3, early imaging should be actively considered rather than adopting a watch-and-wait approach.

More fundamentally, the success of a colorectal anastomosis depends primarily on adequate blood supply and the absence of undue tension, and adjunctive measures such as TDTs cannot compensate for deficiencies in these basic surgical principles. Intraoperative assessment of anastomotic integrity should therefore include careful visual inspection, an air-saline leak test after completion of the anastomosis, and, when available, fluorescence-based perfusion assessment such as indocyanine green (ICG) angiography. In the present case, perfusion of both limbs of the anastomosis was confirmed by ICG fluorescence before transection, and an air-saline leak test demonstrated no detectable leakage after anastomosis; accordingly, a primary diverting ileostomy was not created. Device-related factors should therefore be considered only after patient-related risk factors and anastomotic quality have been carefully evaluated.

It is also essential to clarify the physiological rationale for TDT placement. The primary function of a TDT is often described as reducing the intraluminal pressure gradient across the anastomosis by maintaining partial sphincter relaxation, rather than draining intestinal contents. This rationale, however, is not universally accepted and may not translate into a consistent or clinically meaningful reduction in anastomotic leakage risk. Therefore, careful regulation of insertion depth and ongoing positional monitoring are critical for maximizing any potential preventive benefit while minimizing iatrogenic injury.

Although TDT placement is a simple and minimally invasive adjunct, standardized protocols for insertion technique, depth, fixation method, and postoperative monitoring have yet to be established. This case highlights the need for clearer, evidence-based guidelines to balance the recognized benefits of TDTs with the potential for serious device-related complications. In addition, advanced age and comorbidities such as cardiovascular disease, diabetes, chronic pulmonary disease, immunosuppression, and corticosteroid use are well-known contributors to anastomotic failure, and these patient-related factors should be weighed when deciding between a diverting stoma, TDT placement, or a combination of strategies.

This report has several limitations. First, it describes a single case, and thus the findings may not be generalizable to all patients undergoing rectal surgery with TDT placement. Causality between TDT use and perforation cannot be definitively established, and the proposed mechanism should therefore be regarded as plausible but not exclusive. Second, because there is no unified registry for TDT-related adverse events, the true incidence and risk factors remain uncertain. Establishing a national or international database for device-related complications would help facilitate more accurate risk assessment and inform standardized preventive strategies.

## Conclusions

Transanal drainage tubes are used in some institutions as adjuncts intended to reduce anastomotic leakage after low anterior resection, but their preventive effect remains uncertain, and their clinical value is controversial. Although they are generally considered minimally invasive, they may rarely cause severe device-related complications such as bowel perforation. This case illustrates that even when anastomotic perfusion, integrity, and tension appear satisfactory, and a primary diverting ileostomy is not created, excessive insertion depth and insufficient postoperative reassessment of a transanal tube can lead to iatrogenic bowel injury. If transanal drainage tubes are used, careful limitation of insertion depth, early postoperative imaging, and active monitoring of tube position are essential to minimize risk. More fundamentally, adequate perfusion, lack of undue tension, sound anastomotic technique, and consideration of a proximal diverting stoma in high-risk situations remain the cornerstones of colorectal anastomotic safety. Standardized, evidence-based protocols are needed to clarify whether and how transanal drainage tubes should be used as an adjunct without exposing patients to avoidable harm.
